# Prior information for population pharmacokinetic and pharmacokinetic/pharmacodynamic analysis: overview and guidance with a focus on the NONMEM PRIOR subroutine

**DOI:** 10.1007/s10928-020-09695-z

**Published:** 2020-06-13

**Authors:** Anna H.-X. P. Chan Kwong, Elisa A. M. Calvier, David Fabre, Florence Gattacceca, Sonia Khier

**Affiliations:** 1 Pharmacokinetic and Modeling Department, School of Pharmacy, Montpellier University, Montpellier, France; 2 Probabilities and Statistics Department, Institut Montpelliérain Alexander Grothendieck (IMAG), UMR 5149, CNRS, Montpellier University, Montpellier, France; 3 SMARTc group, Inserm, CNRS, Institut Paoli-Calmettes, CRCM, Aix-Marseille University, Marseille, France; 4 Pharmacokinetics-Dynamics and Metabolism (PKDM), Sanofi R&D, Translational Medicine and Early Development, Montpellier, France

**Keywords:** Population pharmacokinetics, Pharmacokinetic-pharmacodynamic, PRIOR, NONMEM, Guidance, Model

## Abstract

**Abstract:**

Population pharmacokinetic analysis is used to estimate pharmacokinetic parameters and their variability from concentration data. Due to data sparseness issues, available datasets often do not allow the estimation of all parameters of the suitable model. The PRIOR subroutine in NONMEM supports the estimation of some or all parameters with values from previous models, as an alternative to fixing them or adding data to the dataset. From a literature review, the best practices were compiled to provide a practical guidance for the use of the PRIOR subroutine in NONMEM. Thirty-three articles reported the use of the PRIOR subroutine in NONMEM, mostly in special populations. This approach allowed fast, stable and satisfying modelling. The guidance provides general advice on how to select the most appropriate reference model when there are several previous models available, and to implement and weight the selected parameter values in the PRIOR function. On the model built with PRIOR, the similarity of estimates with the ones of the reference model and the sensitivity of the model to the PRIOR values should be checked. Covariates could be implemented a priori (from the reference model) or a posteriori, only on parameters estimated without prior (search for new covariates).

**Graphic abstract:**

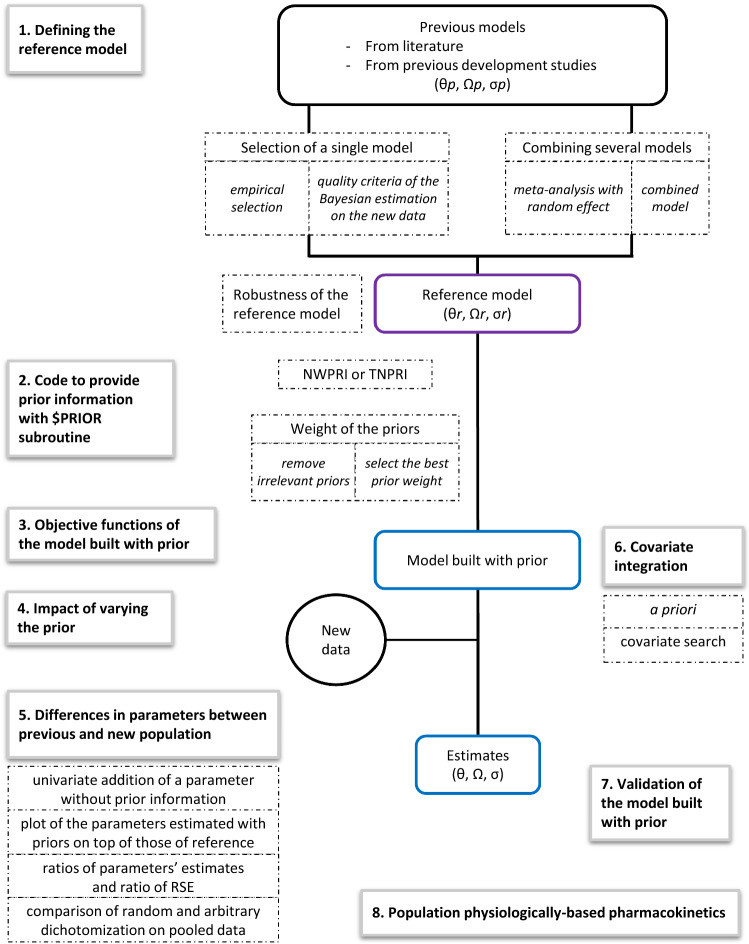

**Electronic supplementary material:**

The online version of this article (10.1007/s10928-020-09695-z) contains supplementary material, which is available to authorized users.

## Introduction

When data are not sufficient to build a model, one may use prior information to stabilize the estimation of some parameters of the model. In population pharmacokinetics (popPK), there are two alternatives to stabilize poorly estimated parameters with prior information: either to fix them to their previous estimated values or to “inform” them thanks to their previous estimated values. “Informing” poorly estimated parameters instead of fixing them reduces the bias in cases where the parameters are slightly different in the previous population and in the population from which the sparse data were collected. To “inform” poorly estimated parameters, the PRIOR subroutine in NONMEM can be used, regardless of the estimation method. Indeed, priors can be included either while using a full Bayesian method (Markov Chain Monte Carlo (MCMC) Bayesian analysis) or a Maximum Likelihood Estimation such as First Order estimation (FO), First Order Conditional Estimation (FOCE), Second Order Conditional Estimation (Laplace) or Expectation Maximization methods (EM methods: Importance Sampling algorithm (IMP) and Stochastic Approximation Expectation Maximization (SAEM)) [1]. Adding a prior to a Maximum Likelihood Estimation would technically convert these into a mode a posteriori (MAP) estimation of the population parameters, even though this term does not show up on the NONMEM report.

Priors are at the heart of Bayesian statistics, whereas they are optional for frequentists [2]. Full Bayesian analysis with “Bayesian” priors places a prior penalty on its conditional likelihood; the same prior penalty is used on maximum likelihood with “frequentist” priors. The OFV is the sum of the OFV on the sparse data (O^S^) and the penalty function (O^P^), which reflects the deviation of the iterated parameters from their previous estimate value [3]. Therefore, it is the sum of O^S^ and O^P^ that is minimized. The main advantage of the “frequentist” priors approach, compared to the “Bayesian” priors approach, is the tremendous decrease in computational time [4].

The pros and cons of the penalty function over simultaneous fitting of all data are similar to those of the Bayesian approach. The main advantages lay in the rapidity and stability of the runs, which is especially important from the industry perspective. One of the disadvantages is the absence of an established robust method for covariate testing when using a penalty function. However, testing for covariates is possible with the penalty function unlike in the Bayesian approach.

While the PRIOR subroutine in NONMEM seems to be a suitable way to analyze sparse or small datasets, literature about the PRIOR subroutine is rare. This review aims at providing a guidance on how to implement and apply the PRIOR subroutine in NONMEM.

## Literature review

### How often is the PRIOR subroutine reported in literature?

Literature was screened for articles reporting the use of the PRIOR subroutine in NONMEM, in a four-step approach, as described in Online Resource 1. In each step, the full text of eligible articles was checked to retain articles actually reporting the use of the PRIOR subroutine in NONMEM. The review of selected articles provided a basis for the guidance developed here. A total of 33 articles reporting the use of the PRIOR subroutine in NONMEM was found in literature [3–35].

### In which context is the PRIOR subroutine used?

One article was methodological and was based on simulations [3]. The 32 other articles analyzed observed sparse data. The methodological article focused on FOCEI (FOCE with eta–epsilon interaction) [3]. Thirty articles analyzing observed data also used Maximum Likelihood Estimation (frequentist approach), all used conditional estimation methods but two used FO because of numerical problems with FOCE [15, 35]. FOCE with the PRIOR subroutine in NONMEM was compared to MCMC Bayesian analysis in WINBUGS for a whole-body physiologically based pharmacokinetic model [4]. Two articles used MCMC Bayesian analysis with $PRIOR NWPRI statement, which allowed the specification of prior parameters distributions [6, 26]. The PRIOR subroutine was mostly used for empirical popPK models in special populations, for example to analyze sparse data from children [9, 10, 12, 13, 18, 20, 25–27] or pregnant women [19, 20, 28]. In one pediatric model, the PRIOR subroutine was used to stabilize the parameters of the maturation function (*i.e.* covariate effects on clearance) to physiologically plausible values, because no data were available for children younger than one year old [20]. Eight articles used the PRIOR subroutine in NONMEM to inform mechanistic popPK models [4, 8, 11, 15, 22, 32–34]. Amongst them, three were Physiologically-Based Pharmacokinetic (PBPK) models [4, 8, 11] (see Sect. 3.8). In all articles, priors were implemented on all pharmacokinetic (PK) parameters or on a subset.

### What are the pros and cons of the PRIOR subroutine?

The main advantages of the PRIOR subroutine are that it can be implemented on a subset of selected parameters using Maximum Likelihood Estimation methods: OMEGA^2^ can be estimated without priors, unlike in Bayesian methods (*e. g.* MCMC Bayesian analysis) [1] and that it runs relatively fast [4]. The PRIOR subroutine is an alternative to fixing the parameters to their previous estimates or to pooling the new data with the previous rich data (when available).

In four studies, the use of priors allowed a better fit of the new data than fixing the parameters [7, 9, 30, 31]. In another study, fixing some parameters led to unrealistic estimates of other parameters, while the use of informative and non-informative (vague) priors on all parameters allowed a correct estimation [12].

In some studies, the use of the prior approach was preferred over pooling sparse with previous data because it allowed the analysis to be completed in one single NONMEM run [15]. Compared to the model built on pooled data, the model built with priors may be more stable and provide a better fit of the new data [27], or reduce the residual unexplained variability [26]. To analyze sparse pediatric data when adult data are available, two articles concluded that it was better to build first the adult model (with allometric scaling) and then use it as prior for the pediatric model than to build a model on pooled data [12, 13]. In the first one, the use of the prior approach prevented the large number of subjects and samples in the rich prior study from driving the estimates of the small new dataset of the population of interest [12]. In the second, the model built on pooled adult and pediatric data was unable to accurately characterize clearance maturation parameters. Indeed, estimations depended on initial estimates and produced large standard errors, probably due to the lack of data in children between 2 and 18 years old [13].

However, implementation of the PRIOR subroutine raises some issues. One article mentioned the PRIOR subroutine but did not retain the prior approach, arguing that the sensitivity to prior information and the assumption of reliability of prior parameterization and structural model may affect the identifiability of the parameters [36].

The articles selected in the present review contain a series of points interesting to consider when using the $PRIOR subroutine. The following guidance summarizes the best reported practices.

## Guidance

### Defining the reference model

Whatever the method used to integrate previous knowledge in a new model, the first step consists in defining the most relevant reference model, with reference parameters that will be implemented as prior (“hyperparameters”). If more than one previous model is available, the reference model may be selected amongst them, using the different criteria presented in Section “Selection of a single model”. Beside model selection, it is possible to combine several models, either in a “combined-model” or using a “meta-analysis with random effects” (Section “Combining several models”). If only one previous model is available, or when one reference model is selected or built, its relevance as the reference model can be assessed with the methods presented in Section “Robustness of the reference model”.

#### Selection of a single model

##### Empirical selection

If several previous models are available, the reference model can be selected amongst them based on (i) population similarity (*e.g.* similar demographic characteristics [6], same geographic region [18]), (ii) the number of relevant estimated structural parameters [12], (iii) the confidence in the estimate(s) of the parameter(s) of interest, according to study design. For example, Kshirsagar et al. wanted to estimate the absorption constant with prior [35]. Their reference model was built on the highest proportion of data (17%) in the early absorption period (up to 2 h), compared to other published models.

##### Quality criteria of the Bayesian estimation on the new data

**Fig. 1:**
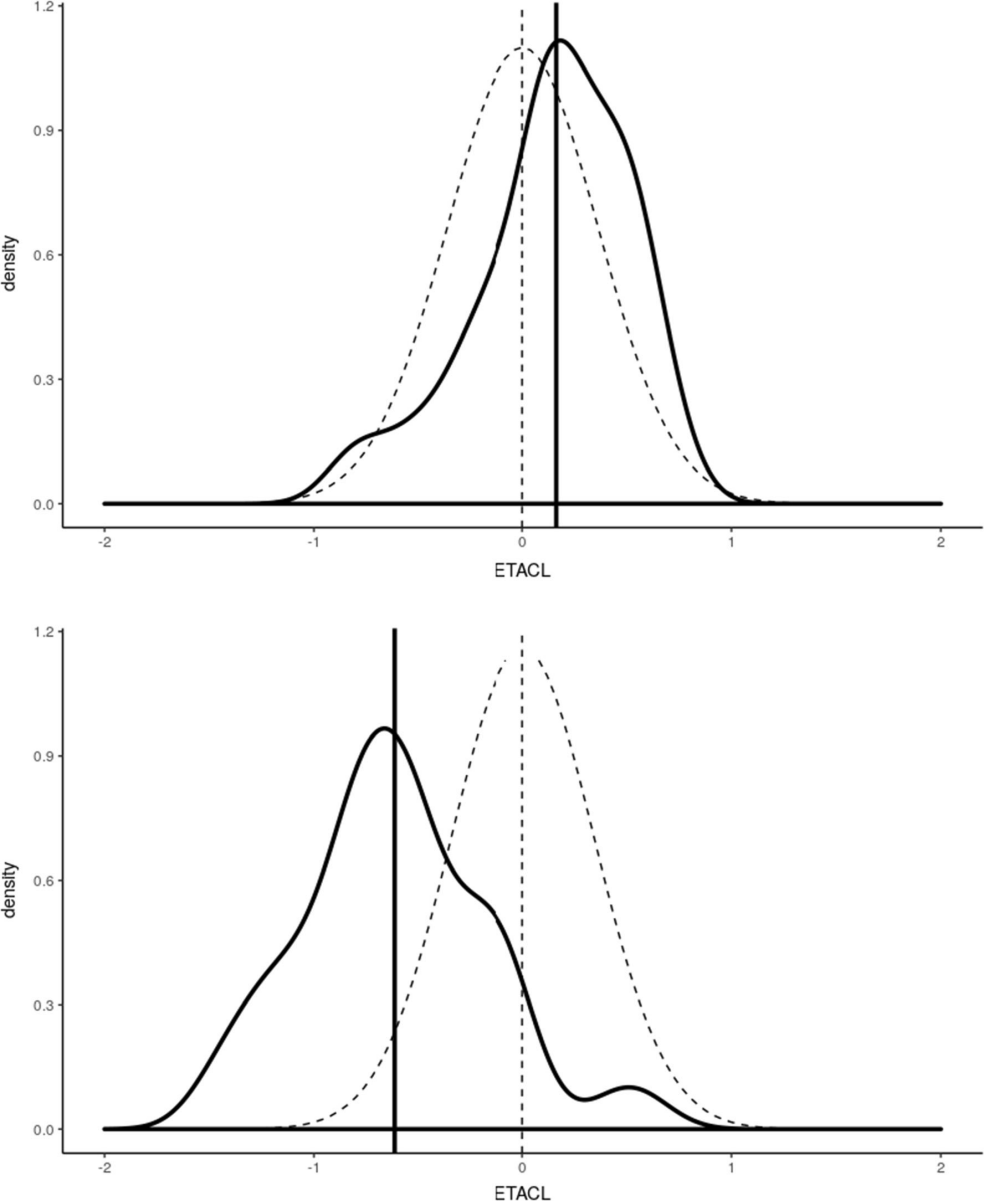
Plot of individual η clearances (black line) on top of theoretical η-distribution N(0, ω^2^) (dotted line). The model resulting in the top plot is to be preferred over the one resulting in the bottom plot. ETACL: η clearances. Adapted from [43]

Knosgaard et al*.* compared literature models regarding their performance as a Bayesian attractor for individual PK parameters estimation from the new data, to choose the most adapted previous model to be used in the PRIOR subroutine [9]. First, a Bayesian estimation of the individual PK parameters for each model was run on the new data (MAXEVAL = 0 in the estimation step allows the estimation of individual η conditional on the initial estimates). Then, the models were ranked by OFV or Akaike Information Criterion (AIC, which applies a penalty to models with more parameters). For each model, the distribution densities of individual η_i_ were compared to the theoretical η-distribution $N$(0,ω^2^). It was hypothesized that the model adequately describes the new dataset if the distributions visually overlap and the η-shrinkage is low, as illustrated in Fig. 1.

The predictive performance of each model may be evaluated by multiple simulations which are then compared to the new data:

- Visual Predictive Checks (VPCs) can be plotted with the new data (external VPC).
- Normalized prediction distribution errors (NPDEs) can be used to compare the simulated concentrations with the observations in the new dataset [37]. The models can then be compared and ranked according to p-values of tests determining whether the NPDEs follow a normal distribution (Wilcoxon sign rank t-test, Fisher test for variance, Shapiro-Wilks test). The best predictive model is the one resulting in the lowest number of tests in which the NPDEs deviate from a normal distribution.

In the systematic comparison of literature models by Knosgaard et al., the predictive performance of the models was more clearly differentiated by NPDEs than VPCs.

#### Combining several models

##### Meta-analysis with random effect

**Fig. 2:**
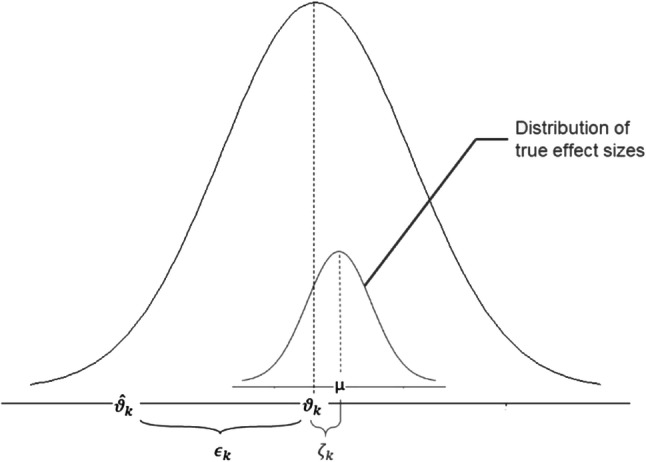
Illustration of parameters of the random-effects-model, from [38]. $\widehat{\theta }$ k = μ + ϵk + ζk (1),$\widehat{\theta }k$: typical value in the study k, μ: typical value in the « universe» of population, ϵk: deviation from the typical value because of sampling errors in the study k, ζk: deviation from the typical value because of over-arching distribution of true effect sizes with the mean, μ, ζk ~ N(μ, τ^2^)

Milosheska et al*.* performed a meta-analysis with random effects to determine the reference parameter values and their uncertainty [23]. In this method, parameter values from structurally identical models are averaged, weighted by their uncertainty. Unlike the meta-analysis with fixed effect, the meta-analysis with random effects assumes that included studies do not come from the same exact population and hypothesizes that there is a distribution of true effect size from a “universe” of populations (**Fig. ****2**). The meta-analysis with random effects can be easily implemented in the R software [38].

##### Combined model

If needed, the reference model can combine models from two (or more) studies having different focus and providing complementary information. Knosgaard et al. analyzed both parent drug and metabolite: they combined the parent drug model and the metabolite model that performed the best in the systematic model comparison stage for each molecule [9]. Brill et al*.* built a model to quantify the interaction effect of antiretrovirals on tuberculosis treatment in patients with both HIV and tuberculosis [7]. The PK parameters of the antituberculosis drug were based on data from two phase IIb studies in subjects without antiretrovirals. The drug interaction effect parameters were based on data from two drug-drug interaction studies in subjects without tuberculosis.

#### Robustness of the reference model

The quality criteria listed in Sect. 3.1.1 can be evaluated on the chosen, built or sole-candidate reference model. External VPCs were also used by Perez-Ruixo et al. to confirm the ability of the allometrically-scaled PK model developed for adults to describe pediatric data [26]. External prediction-corrected VPC (pcVPC) were used by Deng et al*.* and Magnusson et al*.* to verify that the reference model generally fitted the new data [29, 30].

If the previous data are available, one can assess the ability of the reference model to estimate with prior some parameters on a subset of data. Marshall et al*.* used the PRIOR subroutine to build a semi-mechanistic model with sparse data [15]. The reference model included a neutrophil model and a combined PK and receptor model (CD11b receptor). The sparse data contained neutrophil and PK observations but no information on CD11b binding (neither free nor total CD11b measurements), while the model could not be simplified for mechanistic reasons. As the previous data were available, the strength of the previous estimates of CD11b binding parameters was assessable: a model with prior (previous model as prior) was built on the previous data without the observations that allowed the estimation of the CD11b binding data. Robustness was evaluated by assessing the degree of similarity between the estimates of this model and those of the reference model.

In summary, one would ideally select the model which responds best to the objective (*e.g.* characterization of ka). If some models are equivalent as regard to the problematic, it is possible either to use the model which best describes the new data using the Bayesian estimation quality criteria or to build a new model with a meta-analysis. In some cases, the process to be described needs the combination of two or more complementary models that have different focus.

Of note, the reference parameters can be adapted to the target population. For instance, to analyze pharmacokinetics in pregnant women, Lohy Das et al. used a reference model built on both pregnant and non-pregnant women that included pregnancy as a significant covariate on intercompartmental clearance: the reference estimate of intercompartmental clearance was the one calculated with pregnancy effect [19].

Whatever the reference model selected, its robustness should be assessed with a Bayesian estimation on the new data and/or external VPC.

### Code to provide prior information with $PRIOR subroutine

#### NWPRI or TNPRI subroutine?

Two types of PRIOR subroutines can be called: $PRIOR NWPRI or $PRIOR TNPRI, depending on the assumption on the distribution of the prior parameters. Indeed, the prior parameters can be considered normally or Inverse-Wishart distributed [3]. In NWPRI (the most commonly assumed), the fixed parameters THETA are assumed normally distributed and the random parameters OMEGA^2^ (inter-individual and/or inter-occasion variability) are assumed inverse-Wishart distributed. In TNPRI, both are assumed normally distributed.

The methodological article by Gisleskog et al. underlines the theoretical advantage of using TNPRI as compared to NWPRI: in contrast to the normal–inverseWishart distribution (NWPRI), the normal-normal distribution (TNPRI) can express correlations between separate information about the separate values of THETA and OMEGA^2^ [3]. However, in the simulations and tests presented in that article, both methods showed similar percentage of deviation of the parameter estimates and standard errors from their true values.

In our review, only two out of 32 articles analyzing sparse data used TNPRI [15, 29]: in both articles, the previous analysis had been done by the same team. Eighteen articles [4–14, 23, 25, 27, 30, 32–34] used NWPRI; in the remaining 12 articles the method was not specified [16–22, 24, 26, 28, 31, 35], among which six used prior only on THETA [19, 20, 22, 24, 28, 35], the distribution attributed to the OMEGA consequently having no impact.

In practice, the implementation of NWPRI in NONMEM is much simpler than the implementation of TNPRI. In the present version NONMEM 7.4, TNPRI needs an output file from the reference model (msf file) that is not available when using priors from literature.

#### Prior values of the parameters

The prior values to THETA, OMEGA^2^ and SIGMA^2^ should be written and fixed in the control stream in $THETAP, $OMEGAP and $SIGMAP records, respectively. In case of covariances between *n* random components, OMEGA^2^ and SIGMA^2^ matrices should be informed in $OMEGAP BLOCK (*n*) and $SIGMAP BLOCK (n) records.

Although inter-occasion variability is different from inter-individual variability, it is also a random effect coded using OMEGA’s. Thus, prior inter-occasion variability is coded in the same way as prior inter-individual variability.

Usually, the implementation of priors on SIGMA^2^ can be avoided because data contain strong information for estimating the residual error. In most articles, the residual error was estimated independently from the original model. Only two articles used informative priors on SIGMA^2^ [7, 29], without providing rationale for doing so.

Four out of the 33 articles reviewed used log-transformation for the PK model fixed-effect parameters (THETA) [4, 8, 11, 12]. Of note, the three “popPBPK” models (see Sect. 3.8) used this approach to avoid negative sampled values for clearance and tissue affinity [4, 8, 11]. Log-transformation provides stability during the estimation process [12]. When log-transforming THETAs of the reference model, the rules of propagation of errors are used: the variance of the log-transformed THETA is approximately RSE^2, where RSE = SE(THETA)/THETA.

#### Weight of the priors

The weight of each prior into the model is informed by the distribution of the prior parameter. For an assumed normally-distributed prior parameter, the weight is inversely proportional to its variance: the more precise the prior parameter, the more informed the model. When the prior parameter is supposed to be inverse-Wishart distributed, its weight is proportional to its degree of freedom.

Normally distributed parameters (assumed for THETA in NWPRI and for both THETA and OMEGA^2^ for TNPRI) are weighted by their variance–covariance matrix in $THETAPV BLOCK record. When there is only one normally distributed parameter to be weighted, $THETAPV should be used instead of $THETAPV BLOCK. The variance–covariance matrix can be calculated from the SE of the previous model or from a nonparametric bootstrap of this model if the SE are not provided [10]. Large variance sets non-informative priors (e.g. 10^6^ [12]). For informative priors, the full covariance matrix should be preferred. However, this information is not always available and when available, it may lead to minimization issues. In these cases, off-diagonal elements should be set either to 0 [10] or to a very small value (e.g. 10^−7^). Setting off-diagonal elements to zero implies that there is no correlation between the fixed and random effects, which might lead, in theory, to potential bias in model estimates but to date, nothing has been published on this topic.

Inverse-Wishart distributed parameters (assumed for OMEGA^2^ and SIGMA^2^ in NWPRI) are weighted by their degree of freedom in $OMEGAPD and $SIGMAPD records. Their values, as for the normally distributed parameters, depend on the prior informativeness intended. They can range from m + 1, m being the dimension of OMEGA or SIGMA matrix, for uninformative priors, to the number of subjects (for OMEGA) or to the number of observations (for SIGMA) in the previous study for very informative prior. Usually, the degree of freedom for informative OMEGAs is calculated with the formula df = 2*[ OMEGA^2^ / (SE of OMEGA^2^)]^2^ + 1 [1, 6, 11, 23, 39]. The same formula can be applied for informative SIGMAs. The degrees of freedom pertain to the entire OMEGA block, including the off-diagonal elements. Nonetheless it is a general or vague strength parameter, which lends its strength on all elements to the OMEGA block. If a high degree of freedom is placed on an OMEGA prior block, and that block has 0 off-diagonals whereas the data suggests a strong off-diagonal, the analysis may be compromised. If the previous data are available, the degrees of freedom of Inverse-Wishart distribution of OMEGAs can be estimated using maximum likelihood based on the probability density function of the inverse Wishart distribution, for example with R packages mle and diwish [7], or as automatically estimated in Sampling Importance Resampling (SIR) [40]. The aim of SIR is to approximate the true uncertainty of the parameters [41]. Parameters’ vectors are sampled from the covariance matrix, and the model is run on the data with each set of parameters using a Maximum a Posteriori Bayesian estimation (MAXEVAL = 0). When the model is built using the prior subroutine, covariance matrix is taken from the previous model. Then, parameters are resampled according to an importance ratio computed thanks to the previous step. This resampling is repeated. And then, for each OMEGA, an inverse-Wishart distribution can be fitted to the distribution of the resampled OMEGA: the degree of freedom of the inverse-Wishart distribution is the one that can be reported in $OMEGAPD.

**Fig. 3:**
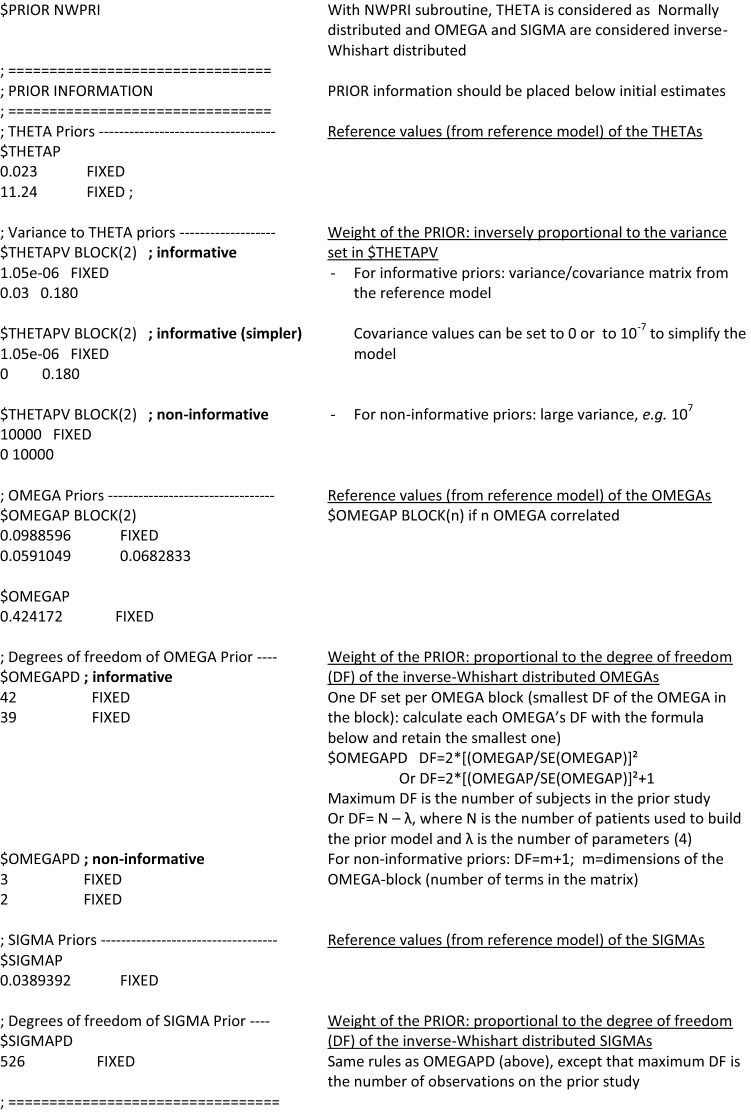
Example of codes of a NONMEM control file for implementation of PRIOR, NWPRI subroutine, informative and non-informative priors; as defined by Bauer [1] and Gisleskog et al*.* [3]

Figure 3 summarizes how to code the prior weight in the control file. Non-informative distribution can also be referred to as vague [12], because as long as a prior is used, it remains at least slightly informative.

Eight out of 32 articles analyzing sparse data reviewed implemented informative priors on all parameters [5, 10, 12, 15, 23, 29–31], 18 implemented informative priors, and/or so-called “weakly informative” (when they are associated to 10% [20] or 50% [24, 28] uncertainty) only on a part of the parameters [7–9, 13, 16–22, 24, 25, 28, 32–35], and three implemented uninformative priors on some of the parameters while had informative priors on the rest of the parameters [4, 6, 11]. The latter included the study using MCMC [6] and the study comparing FOCE to MCMC [4]. Three articles did not precise how informative the priors were [14, 26, 27].

When only a subset of the parameters is estimated with prior information, these parameters should be the first declared in NONMEM: the first n parameters, where n is the number of parameters defined in the $THETAP and/or $OMEGAP statement, will have priors, and the following parameters will not.

Reducing the prior weight and even suppressing them is useful to obtain the most information from the new population. A covariate search should be conducted only on the parameters estimated without prior (see Sect. 3.6). Moreover, if a model with full informative priors has much greater estimates of interindividual variability as compared to the prior value, it might stem from the strength of the prior values for the corresponding fixed effects together with a potentially different population parameter estimate in the new population. In this case, it seems interesting to reduce the prior weight or to remove the prior from these parameters (from both THETA and OMEGA if possible) [7].

##### Approaches to remove priors

To determine if a parameter can be estimated without prior, the ratio of RSE of PK parameters estimates from the model built with prior to the RSE from the previous model can be used: if the RSE ratio is very small, one can consider deleting the prior on the corresponding parameter [15] (see the approach of Marshall et al*.* in Sect. 3.5).

Stevens et al*.* removed the priors from each parameter in turn. For each re-estimation, they observed the impact on the OFV and on value and precision of other parameters [34]. They tested the priors on two parameters and decided to keep the priors on both: together with a drop of OFV, the remaining parameter had plausible values and smaller confidence intervals. For example, when they removed the prior on the drug’s EC50 (concentration that induces half the maximum effect Emax), the Emax estimate increased threefold and was less plausible.

Knosgaard et al*.* also tested different combinations of priors (*e.g*. prior on THETA, with or without prior on OMEGA) to select the one that gives the lowest OFV [9].

##### Approaches to select prior weight

To implement the best weight of priors, Magnusson et al*.* compared the results of models with informative priors weighted on the one hand by an assigned 10% uncertainty and on the other hand by their smaller reference uncertainties, in terms of VPC (see Fig. 2 in the original article [30]), residual unexplained variability, inter-individual and inter-occasion variability parameters. In this case, the model with 10% uncertainties assigned to the model parameters provided the most adequate description of the data and decreased variability parameters.

In their pediatric model, Knebel et al. varied the informativeness of the adult priors so as to minimize the influence of adult prior information but still allow a stable estimation: variance was set to 10^6^ for half of the THETAs (uninformative) and degree of freedom of OMEGAs were fixed to the smallest value possible (the dimension of the OMEGA matrix) [12].

Krogh-Madsen et al. also tested different values of degree of freedom for OMEGA: there were only little changes in the parameter estimates. Therefore, the degree of freedom in the final model was set to the lowest possible value, considering it more appropriate to compensate for the choice of distribution (i.e*.* inverse Wishart) [14].

To sum up, NWPRI can be preferred over TNPRI, unless there is a strong correlation between THETA and OMEGA. Priors on SIGMA^2^ should be avoided. Different combinations and weights of priors should be performed to select the one that performs best, i.e. give the lowest OFV, confidence intervals, residual variability, and have the best predictive ability with VPC.

### Objective functions of the model built with prior

The NONMEM output file displays two blocks of information for OFV for models built with prior.

The first is the same as the one obtained with models without prior:

- TOTAL DATA POINTS NORMALLY DISTRIBUTED (N)
- N*LOG(2PI) CONSTANT TO OBJECTIVE FUNCTION
- OBJECTIVE FUNCTION VALUE WITHOUT CONSTANT: objective function on the data, including the prior penalty (usually reported) = *O*^*S*^ + *O*^*P*^
- OBJECTIVE FUNCTION VALUE WITH CONSTANT: sum of the two terms above

The second is specific to models built with prior:

- PRIOR CONSTANT TO OBJECTIVE FUNCTION: constants pertaining to wisharts of OMEGAs, SIGMAs, and normal of THETAs (appropriate multiple of LOG(2PI))
- OBJECTIVE FUNCTION VALUE WITHOUT CONSTANT: objective function on the data, including the prior penalty (the same as the one in the first block) = *O*^*S*^ + *O*^*P*^
- OBJECTIVE FUNCTION VALUE WITH *(PRIOR)* CONSTANT: sum of the two terms above

The objective function with constant is used only for compatibility with how other software may report the OFV. The prior contribution on the objective function (prior penalty, O^P^) is included in the reported OFVs. The OFV on the data (O^S^) is the sum of the individual OFV reported in the phi file of NONMEM outputs. O^S^ is the OFV calculated with the tweaked model (model where initial estimates were set to final estimates of the model built with priors) run with MAXEVAL = 0 NOPRIOR = 1 (without prior). O^P^ can then be calculated as the difference between the reported OFV without constant and O^S^.

Once the prior is used in the base model, the total OFV (which includes the prior penalty) can be used in the Likelihood Ratio Tests (LRT), if no change is made to the prior information. Thus, O^S^ should not be used in the LRT, as the prior was involved in the original fitting so O^S^ is not a minimum OFV by itself (not a maximum likelihood position).

Therefore, the OFV to be used in comparisons using LRT is the total OFV that is reported in the output of NONMEM (OFV without constant = O^S^ + O^P^).

### Impact of varying the prior

Milosheska et al. tested the sensitivity of the model parameters [23] (i) to the prior specification by varying the prior values by − 50% and + 50%, and ii) to the informativeness of the prior by changing the precision of the prior (SE from − 50% to + 50%)*.* The impact of changing prior values and precision was quantified by the resulting change in the estimate of the impacted parameter. The sensitivity of the model to the weight of the prior was considered acceptable as parameter estimates remained within ± 15% range when the SE varied of ± 50%. If changing the prior value results in identical change in parameter estimates, it means that the new data contains little information about this parameter: the prior is important in the model and it should be carefully defined and trusted.

Lledo-Garcia et al. also varied the precision of all priors (simultaneously made less informative by increasing their associated variances tenfold): the change was lower than 6% in each parameter estimate and was thus qualified as “very minor” [33].

Denti et al. tested different settings for the prior distribution to show that the estimates of the other parameters in the model were not significantly affected [24].

To date, there is no standardized method to quantify the impact on the model of varying the value and weight of the prior. However, it is recommended to quantify both the change in the estimate of the impacted parameter and the stability of other parameters when the prior value and weight are modified.

### Differences in parameters between previous population and new population

The parameters implemented with priors should be similar in the previous and the new population. Otherwise, the new estimates would be constrained to a biased value, leading to a misfit of the model built with prior. Moreover, the estimation of other parameters could also be impacted. Pharmacometricians used different strategies to verify the hypothesis of similarity between parameters in the previous and in the new populations. Some of these strategies can also be used to characterize the amount of information given by the new (sparse and/or small) dataset compared to the prior information.

Brill et al*.* tested whether the univariate addition of a parameter without prior information showed any significant improvement in OFV for each parameter of the model built with full prior [7], that is, testing on each parameter if the addition of a parameter of difference (*e.g.* named DIS), estimated on the new data without prior, significantly improved the OFV of the model built with prior. For each parameter, two models are compared with the LRT: one with the parameter estimated with prior (DIS = 0), one with the parameter estimated with prior multiplied by (1 + DIS). This can be done with automated Stepwise Covariate Modelling (SCM) in PsN® [40]: the difference in parameters between the previous data and the new data can be coded DIS = 1, reflecting the difference between the populations of the two datasets [42]. The difference in OFV between the two models should be compared to an actual significance level that can be computed with Stochastic Simulation and Estimation (SSE) in PsN®. If the addition of DIS (estimated on the data only) on a parameter significantly improves the OFV, the parameter differs between previous population and new population. It is then more appropriate to either remove the prior from this parameter or take DIS into account. A code for the SCM configuration file and the SSE command for the current situation is proposed in Online Resource 2. Similarly, Chotsiri et al. investigated inter-study differences between the new and the prior study by applying a categorical study covariate on all pharmacokinetic parameters [18]. The body weight-normalized exposure was lower in children 2 months to 5 years old from the new study than in the older children from the reference model. As the data collected in the new study were not sufficient to explain this discrepancy, a categorical “study” covariate was applied to the relative bioavailability. Physiological explanations were only hypothesized.

**Fig. 4:**
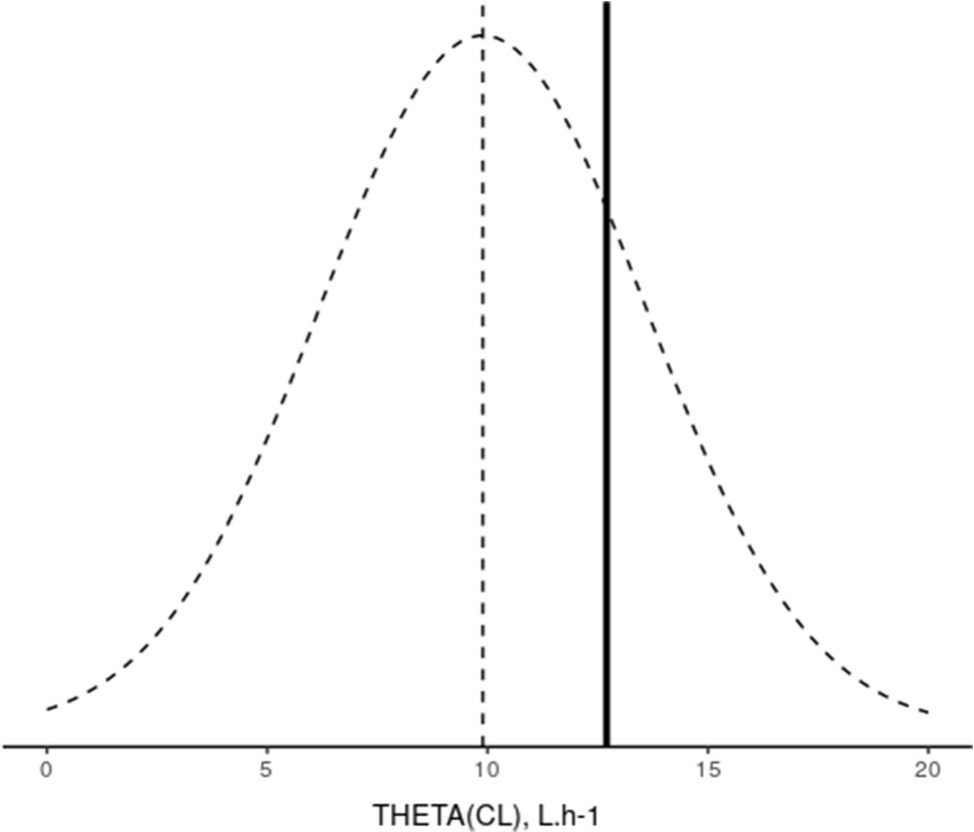
Case example of the plot proposed by Tsamandouras et al*.* [11], data from [43]. The estimate of THETA(CL) in the model built with prior was 12.7 L.h^−1^ (black line). THETA(CL) in the reference model was 9.89 L.h^−1^ and its standard deviation 3.71 L.h^−1^ (dotted lines)

Tsamandouras et al. proposed to plot the estimates of the parameters estimated with priors on top of the distributions representing the available prior knowledge (prior uncertainty in a population model parameter), to visualize the degree to which these estimates were tweaked from the priors [11] (Fig. 4).

Marshall et al*.* proposed an approach based on ratios of parameters’ estimates and RSE [15]. They compared each PK parameter estimate from the model built with prior over the one from the previous model and identified three cases:

- Ratio of the parameter estimate ~ 1 and ratio of the corresponding RSE ~ 1: the sparse data does not provide information on this aspect of the model
- Ratio of the parameter estimate ~ 1 and ratio of the corresponding RSE < 1: the sparse data adds information on this parameter
- Ratio of the parameter estimate ≠ 1 (ratio of the corresponding RSE should be >  > 1): the parameter differs between the two populations; in this case the parameter should not be estimated with priors.

This method should be applied with caution. Lledo-Garcia et al*.* investigated a lack of decrease in uncertainty in one parameter compared to the prior (ratio of RSE close to unity) [33]. They fixed the parameter that had a marked reduction in uncertainty and estimated LS (life span), the parameter that had no decrease in uncertainty: LS was well estimated without prior (low RSE). This illustrated that the data did contain information about LS. Moreover, LS estimate was close to the reference value: this information was in agreement with the prior value.

To compare the pharmacokinetic parameters distributions between adults and children, Perez-Ruixo et al. used a “parametric bootstrap approach” on a dataset of 12 children (12 samples per children) [26]. They compared the estimates of the model built with prior to the theoretical distribution of parameters that would have been obtained if adults and children had the same parameter distributions. This theoretical distribution was obtained with stochastic simulation (with uncertainty) and estimation. First, they simulated 1000 (new) pediatric datasets with uncertainty, using the parameters distributions of a nonparametric bootstrap of the (reference) adult model with allometric scaling. Then, they estimated the parameters of the model built with prior on each of the 1000 simulated datasets. The distribution of these estimated parameters constituted the theoretical distribution. The estimates of the fixed effect parameters of the model built with prior was within the 95% confidence interval of the theoretical distribution, which confirmed the similarity of the pharmacokinetic parameter point estimates between adults and children. On the contrary, the estimates of the between-subjects and residual variability of the model built with prior were out of the 95% confidence interval of the theoretical distribution, which did not confirm the similarity of PK parameter distribution between adults and children. The authors proposed this approach to detect differences in the distribution of PK parameters between adults and children. However, this approach is questionable, since the prior constrains the estimation of pediatric parameters to be similar to the adult estimates. If there were differences in PK parameters between the children and the adult dataset, it would be difficult to find them as the parameters estimates on the children dataset are already constrained by the adult values. In that article, what is interpreted by the authors as a difference in PK parameter distribution (OMEGA) could actually be a difference in PK parameters compensated by an inflated parameter distribution due to the constrained bias in PK parameter estimates.

In cases where the model on new data can be built without prior (*e.g.* priors are used to stabilize the model and to avoid flip-flop kinetics), its final parameter values can be compared with those of the model built with prior [14, 35].

In cases where the previous data are available, the similarity in PK parameter distribution between populations can be assessed by comparing the results of models estimated without prior on the pooled data stratified by two different approaches [27]. The first stratification is an arbitrary dichotomization that stratifies the pooled dataset by population (previous and new populations). The second stratification is a random dichotomization implemented by the MIXTURE subroutine in NONMEM, which identifies two subpopulations with different PK parameters. If the previous and the new populations are identified as subpopulations in the random dichotomization, the previous and the new populations are different: the model on the new population should not integrate prior from the previous population. If the subpopulations identified in the random dichotomization are not consistent with the arbitrary split, *i.e.* there are individuals from both the previous and the new population in each subpopulation, it is assumed that previous and new data are part of the same population: the variability in PK parameters can be described by covariates. In this case, it is possible to estimate on the new data a model built with prior from the previous population.

Altogether, the choice of the method to assess the differences in parameters between previous and new populations depends on the constraints of the analysis. If time is not an issue, testing the new study as a categorical covariate on all pharmacokinetic parameters would be the most recommended approach because it is reproducible thanks to the automatization in PsN. When previous data are available, the comparison of arbitrary and random stratifications is simpler.

### Covariate integration

#### Implementation a priori

Covariates of the reference model can be included a priori in the model built with prior, especially if there is a strong belief that they are the same in the previous and the new population. For example, besides allometric scaling, scaling factors for preterm neonates are supposed to remain the same: one can prefer to fix the scaling factor values to their previous estimates, and to estimate the standardized parameter with or without prior [9]. However, the risk of over-parameterizing the model by introducing covariates based on assumptions should be taken into account.

Priors may be implemented only on covariate effects. Ali et al*.* used the PRIOR subroutine to stabilize only the parameters of the maturation function (covariate effects on clearance) to physiologically plausible values, because no data were available for children younger than one year old [20]. Similarly, in their PKPD model of artesunate and dihydroartemisinin, Lohy Das et al. used priors to include the effect of declining parasite densities (*i.e.* malaria disease effect) on pharmacokinetic parameters, as concentration measurements were absent after the first dose [21].

#### Assessment of the impact of covariates implemented a priori

After implementation a priori, the impact of covariates can be assessed in terms of effect on the parameter, for example graphically plotting parameter versus covariate [7, 9, 12]. The physiological plausibility has to be taken into account. To test if the covariate is still significantly influencing the model on the new population, it is unclear how the OFV can be used for comparison between models: the LRT cannot be used to compare models directly with ∆OFV when changes are made in the PRIOR information [14].

Krogh-Madsen et al*.* chose to compare OFV on the data (O^S^). The two models built with prior (one using the previous model without covariate and the other using the previous model with one covariate) were run on the new dataset. Their parameters estimates were then fixed to run the same two “tweaked” models without re-estimation (MAXEVAL = 0) on the same dataset. The OFVs were compared using the LRT. However, as stated in Sect. 3.3, O^S^ should not be compared between nested models when prior information was used to minimize the OFV.

#### Search for new covariates

##### Stepwise covariate modelling

Stepwise covariate modelling is questionable when using the PRIOR subroutine. In some articles, covariates were added on parameters estimated with priors using the classical stepwise covariate modelling with forward inclusion and backward deletion (for example, p < 0.05, that is a threshold of ∆OFV = 3.84 in the hypothesis that the ∆OFV is Chi-square distributed) [5, 19, 23]. In practice, specific considerations should be kept in mind when searching for covariates on parameters that are estimated with priors:

- with prior information on THETA, the typical values of the parameters are constrained to be close to the one of the reference model: if the covariates were not similarly distributed in the previous and the new population, the covariate should be centered around its median in the previous dataset. Alternatively, the median covariate of the new dataset could be used, but the THETAP shall be adjusted accordingly, and it is important to take into account that the uncertainty of the parameter, which depends on the normalization, can be biased.
- with prior information on ETA, the introduction of a covariate would decrease less the inter-individual variability than if the inter-individual variability was estimated on the new dataset.

As much as possible, covariate search on model parameters estimated with priors should not be performed: if the new data is statistically too weak to support a PK/PD parameter, even on a base model, then the statistical power is likely too low to support a covariate analysis on that parameter. Instead, one should rather search for covariates only on parameters without prior [44]. In a first step, the priors can be removed from all parameters that can be estimated without prior (see ‘Approaches to remove the priors’ in Sect. 3.2.3). Subsequently, the covariate search can be performed on these parameters.

##### Full covariate modelling

Robbie et al*.* used a full covariate modelling approach [13]. Estimates of covariate effects were examined in the context of magnitude of effect and precision of effect size. Covariates were kept if the 95%CI of their estimates given by the bootstrap did not include 1 (equivalent to no effect). The covariate modelling approach, which emphasized parameter estimation rather than stepwise hypothesis testing, was used for this population PK analysis to avoid issues associated with the likelihood ratio test in mixed-effect models, including correlation or co-linearity of predictors, multiple comparisons, and artificial parameter precisions.

### Validation of the model built with priors

Most articles reviewed validated the models built with priors using simulation-based diagnostics (i.e. VPC [5, 6, 9, 11–14, 17–24, 28, 30–34], pcVPC [8, 10, 16, 29], and NPDE [9, 25, 29]). Some used bootstrap [4, 5, 9, 11–14, 16–18, 20, 23–25, 27, 32, 34], SIR [28] and external validation [30, 33, 34].

It is important to underline that simulation with a model built with priors does take the priors into account to simulate with uncertainty. To simulate without uncertainty, one must turn off the prior (that represents the population parameter uncertainty) in the tweaked model. This is performed by simply removing the priors from the simulation model file. This is the case if the aim is to verify that the final estimates adequately describe the data. The uncertainty of the population parameters can be included to cover a wide range of possibilities if the goal is to investigate all the possible datasets that can occur on a future trial. This functionality can be used on a simple model built without prior, with the specification of the uncertainty in the $PRIOR positions.

### Population Physiologically-Based PK (popPBPK)

In three articles, the models implemented as priors were Physiologically-Based Pharmacokinetic (PBPK) models, since they included two types of input data: system-related (physiological) parameters (*e.g.* blood flows, organ volumes, tissue compositions) and drug-related parameters (*e.g.* plasma protein binding, clearance and plasma to tissue partition coefficients (Kp)).

Over these three, two were “whole-body physiologically-based PK model (WBPBPK)” [4, 8], and one was a so-called “mechanistic PK model using an integrated popPBPK approach” [11], which included a compartment “rest of the body”. In these PBPK models, the system-related parameters were fixed, as they were considered known at the fixed effect (typical individual) level. Besides, the drug-related parameters were estimated with priors, as these parameters were informed from in vitro experiments or in silico calculation and were therefore associated with a certain degree of inaccuracy/imprecision. Depending on the model, interindividual variability was estimated for clearance and/or Kp value(s).

Compared to a full Bayesian analysis in WINBUGS, run time was substantially shortened and estimates were similar [4]. Moreover, unlike the full Bayesian analysis, the PRIOR functionality allowed to estimate some of the parameters without prior, which comes in handy when prior information is missing for some of the model parameters [11].

## Conclusion

The PRIOR subroutine is a valuable approach to analyze sparse/rare data or estimate mechanistic-based models in an easy way and acceptable run times. Even if this subroutine has been available in NONMEM for years, its use is still uncommon: few articles are available and a lot of questions remains. Anyway, some recommendations can ease the future use of this function and limit the risk of misuse. First of all, the choice the reference model is critical: it can be either carefully selected or combined from several models. Regardless of the reference model, it is best to test the robustness of the final model, e.g. with external VPC. In order to specify the prior weight, the usual approach is to retain the model with priors on the least parameters and with the lowest informativeness on some parameters, but that still allows for a good estimation. The sensitivity of the model parameters to the prior specification should also be evaluated. Then, it is mandatory to quantify the differences in parameters between previous and new populations. If significant differences are detected, it means that the prior constrains the estimate to a biased value, which should be taken into account in the analysis. Finally, it is tricky to identify new covariates or to confirm previously existing parameter/covariate relationships with a model built with prior. Covariate search should be avoided on parameters estimated with priors.

## Electronic supplementary material

Below is the link to the electronic supplementary material.

Supplementary file1 (DOCX 71 kb)

Supplementary file2 (SCM 3 kb)

## Abbreviations

FOCE: First Order Conditional Estimation
FOCEI: First Order Conditional Estimation with Interaction
IMP: Importance sampling algorithm
LRT: Likelihood Ratio Test
NONMEM: NON-linear Mixed Effect Modelling
NPDE: Normalized Prediction Distribution Errors
NWPRI: Normal Inverse-Wishart Prior
OFV: Objective Function Value
O^P^: Penalty function on the OFV
O^S^: OFV on the sparse data
pcVPC: Prediction corrected VPC
PKPD: PharmacoKinetic-PharmacoDynamic
PopPK: Population PharmacoKinetics
RSE: Relative Standard Error
SAEM: Stochastic Approximation Expectation Maximization
SCM: Stepwise Covariate Modelling
SE: Standard Error
SIR: Sampling Importance Resampling
TNPRI: Normal-Normal Prior
VPC: Visual Predictive Checks
