# Supplementary material for: Prior information for population pharmacokinetic and pharmacokinetic/pharmacodynamic analysis: overview and guidance with a focus on the NONMEM PRIOR subroutine
Source: J Pharmacokinet Pharmacodyn. 2020 Jun 13;47(5):431–46. doi: 10.1007/s10928-020-09695-z (PMC7520416; doi:10.1007/s10928-020-09695-z)
Supplement: Supplementary file 1 — Supplementary file1 (DOCX 71 kb) [file 10928_2020_9695_MOESM1_ESM.docx]

The selection procedure:

1. First, a systematic electronic database search was performed in PubMed from inception to March 2020 with the keywords (PRIOR[All Fields] AND NONMEM[All Fields]) OR (PRIORS[All Fields] AND NONMEM[All Fields]). Ninety-six articles were found, of which 12 reported the use of the PRIOR subroutine in NONMEM [1–12].
2. Second, the references of the articles selected in the first step were screened for any methodological article about the PRIOR subroutine. Only one methodological article was found [13].
3. Third, the articles citing the methodological article found in the second step were checked in the PubMed database. Forty-two articles were found, of which 18 reported the use of the PRIOR subroutine in NONMEM but had not been detected in the first step [14–31].
4. Additionally, backward reference search was performed on the articles selected in steps 1, 2 and 3 to identify additional articles that met the selection criteria. Two more articles were found [32, 33].

Figure 1: Selection procedure for the 33 articles considered in the current review, with n = number of selected or removed articles at each step or sub-step.


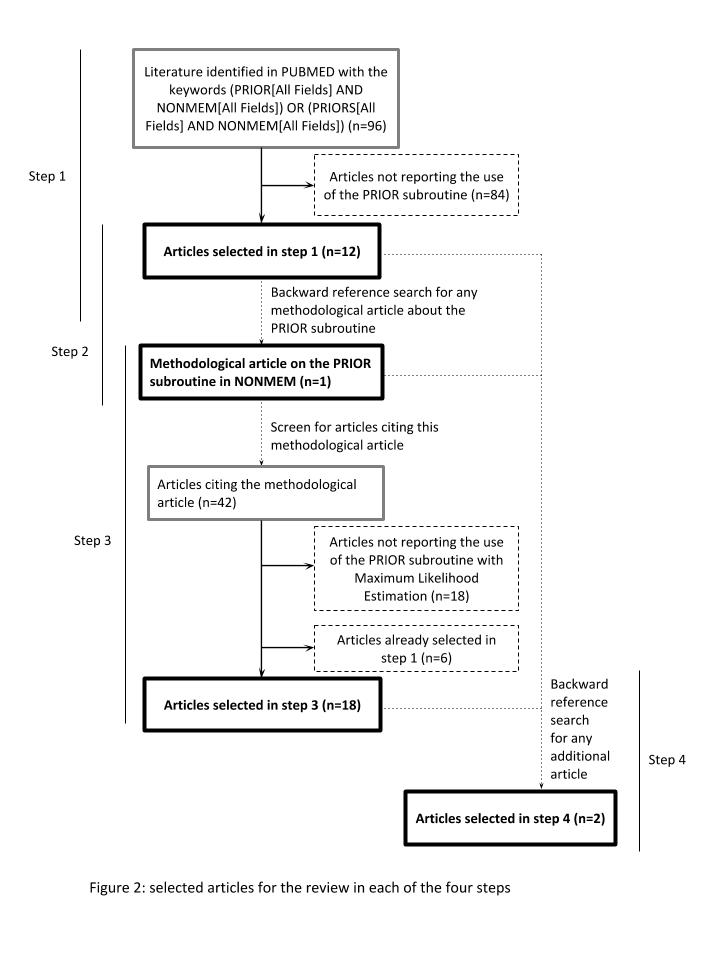


REFERENCES

1. Saito M, Kaibara A, Kadokura T, Toyoshima J, Yoshida S, Kazuta K, Ueyama E (2019) Pharmacokinetic and pharmacodynamic modelling for renal function dependent urinary glucose excretion effect of ipragliflozin, a selective sodium–glucose cotransporter 2 inhibitor, both in healthy subjects and patients with type 2 diabetes mellitus. British Journal of Clinical Pharmacology 85:1808–1819 . https://doi.org/10.1111/bcp.13972

2. Nemoto A, Masaaki M, Yamaoka K (2017) A Bayesian Approach for Population Pharmacokinetic Modeling of Alcohol in Japanese Individuals. Curr Ther Res Clin Exp 84:42–49 . https://doi.org/10.1016/j.curtheres.2017.04.001

3. Brill MJE, Svensson EM, Pandie M, Maartens G, Karlsson MO (2017) Confirming model-predicted pharmacokinetic interactions between bedaquiline and lopinavir/ritonavir or nevirapine in patients with HIV and drug-resistant tuberculosis. Int J Antimicrob Agents 49:212–217 . https://doi.org/10.1016/j.ijantimicag.2016.10.020

4. Sadiq MW, Nielsen EI, Khachman D, Conil J-M, Georges B, Houin G, Laffont CM, Karlsson MO, Friberg LE (2017) A whole-body physiologically based pharmacokinetic (WB-PBPK) model of ciprofloxacin: a step towards predicting bacterial killing at sites of infection. J Pharmacokinet Pharmacodyn 44:69–79 . https://doi.org/10.1007/s10928-016-9486-9

5. Knøsgaard KR, Foster DJR, Kreilgaard M, Sverrisdóttir E, Upton RN, van den Anker JN (2016) Pharmacokinetic models of morphine and its metabolites in neonates:: Systematic comparisons of models from the literature, and development of a new meta-model. Eur J Pharm Sci 92:117–130 . https://doi.org/10.1016/j.ejps.2016.06.026

6. Muto C, Shoji S, Tomono Y, Liu P (2015) Population Pharmacokinetic Analysis of Voriconazole from a Pharmacokinetic Study with Immunocompromised Japanese Pediatric Subjects. Antimicrob Agents Chemother 59:3216–3223 . https://doi.org/10.1128/AAC.04993-14

7. Tsamandouras N, Dickinson G, Guo Y, Hall S, Rostami-Hodjegan A, Galetin A, Aarons L (2015) Development and Application of a Mechanistic Pharmacokinetic Model for Simvastatin and its Active Metabolite Simvastatin Acid Using an Integrated Population PBPK Approach. Pharm Res 32:1864–1883 . https://doi.org/10.1007/s11095-014-1581-2

8. Knebel W, Gastonguay MR, Malhotra B, El-Tahtawy A, Jen F, Gandelman K (2013) Population pharmacokinetics of atorvastatin and its active metabolites in children and adolescents with heterozygous familial hypercholesterolemia: selective use of informative prior distributions from adults. J Clin Pharmacol 53:505–516 . https://doi.org/10.1002/jcph.66

9. Robbie GJ, Zhao L, Mondick J, Losonsky G, Roskos LK (2012) Population pharmacokinetics of palivizumab, a humanized anti-respiratory syncytial virus monoclonal antibody, in adults and children. Antimicrob Agents Chemother 56:4927–4936 . https://doi.org/10.1128/AAC.06446-11

10. Krogh-Madsen M, Bender B, Jensen MK, Nielsen OJ, Friberg LE, Honoré PH (2012) Population pharmacokinetics of cytarabine, etoposide, and daunorubicin in the treatment for acute myeloid leukemia. Cancer Chemother Pharmacol 69:1155–1163 . https://doi.org/10.1007/s00280-011-1800-z

11. Langdon G, Gueorguieva I, Aarons L, Karlsson M (2007) Linking preclinical and clinical whole-body physiologically based pharmacokinetic models with prior distributions in NONMEM. Eur J Clin Pharmacol 63:485–498 . https://doi.org/10.1007/s00228-007-0264-x

12. Marshall S, Macintyre F, James I, Krams M, Jonsson NE (2006) Role of mechanistically-based pharmacokinetic/pharmacodynamic models in drug development : a case study of a therapeutic protein. Clin Pharmacokinet 45:177–197 . https://doi.org/10.2165/00003088-200645020-00004

13. Gisleskog PO, Karlsson MO, Beal SL (2002) Use of Prior Information to Stabilize a Population Data Analysis. J Pharmacokinet Pharmacodyn 29:473–505 . https://doi.org/10.1023/A:1022972420004

14. Golubović B, Vučićević K, Radivojević D, Kovačević SV, Prostran M, Miljković B (2019) Exploring Sirolimus Pharmacokinetic Variability Using Data Available from the Routine Clinical Care of Renal Transplant Patients - Population Pharmacokinetic Approach. J Med Biochem 38:323–331 . https://doi.org/10.2478/jomb-2018-0030

15. Naidoo A, Chirehwa M, Ramsuran V, McIlleron H, Naidoo K, Yende-Zuma N, Singh R, Ncgapu S, Adamson J, Govender K, Denti P, Padayatchi N (2019) Effects of genetic variability on rifampicin and isoniazid pharmacokinetics in South African patients with recurrent tuberculosis. Pharmacogenomics 20:225–240 . https://doi.org/10.2217/pgs-2018-0166

16. Chotsiri P, Zongo I, Milligan P, Compaore YD, Somé AF, Chandramohan D, Hanpithakpong W, Nosten F, Greenwood B, Rosenthal PJ, White NJ, Ouédraogo J-B, Tarning J (2019) Optimal dosing of dihydroartemisinin-piperaquine for seasonal malaria chemoprevention in young children. Nat Commun 10:480 . https://doi.org/10.1038/s41467-019-08297-9

17. Lohy Das J, Rulisa S, de Vries PJ, Mens PF, Kaligirwa N, Agaba S, Tarning J, Karlsson MO, Dorlo TPC (2018) Population Pharmacokinetics of Artemether, Dihydroartemisinin, and Lumefantrine in Rwandese Pregnant Women Treated for Uncomplicated Plasmodium falciparum Malaria. Antimicrob Agents Chemother 62: . https://doi.org/10.1128/AAC.00518-18

18. Ali AM, Penny MA, Smith TA, Workman L, Sasi P, Adjei GO, Aweeka F, Kiechel J-R, Jullien V, Rijken MJ, McGready R, Mwesigwa J, Kristensen K, Stepniewska K, Tarning J, Barnes KI, Denti P (2018) Population Pharmacokinetics of the Antimalarial Amodiaquine: a Pooled Analysis To Optimize Dosing. Antimicrob Agents Chemother 62: . https://doi.org/10.1128/AAC.02193-17

19. Lohy Das JP, Kyaw MP, Nyunt MH, Chit K, Aye KH, Aye MM, Karlsson MO, Bergstrand M, Tarning J (2018) Population pharmacokinetic and pharmacodynamic properties of artesunate in patients with artemisinin sensitive and resistant infections in Southern Myanmar. Malar J 17: . https://doi.org/10.1186/s12936-018-2278-5

20. Guiastrennec B, Sonne D, Hansen M, Bagger J, Lund A, Rehfeld J, Alskär O, Karlsson M, Vilsbøll T, Knop F, Bergstrand M (2016) Mechanism‐Based Modeling of Gastric Emptying Rate and Gallbladder Emptying in Response to Caloric Intake. CPT Pharmacometrics Syst Pharmacol 5:692–700 . https://doi.org/10.1002/psp4.12152

21. Milosheska D, Lorber B, Vovk T, Kastelic M, Dolžan V, Grabnar I (2016) Pharmacokinetics of lamotrigine and its metabolite N‐2‐glucuronide: Influence of polymorphism of UDP‐glucuronosyltransferases and drug transporters. Br J Clin Pharmacol 82:399–411 . https://doi.org/10.1111/bcp.12984

22. Denti P, Jeremiah K, Chigutsa E, Faurholt-Jepsen D, PrayGod G, Range N, Castel S, Wiesner L, Hagen CM, Christiansen M, Changalucha J, McIlleron H, Friis H, Andersen AB (2015) Pharmacokinetics of Isoniazid, Pyrazinamide, and Ethambutol in Newly Diagnosed Pulmonary TB Patients in Tanzania. PLoS One 10: . https://doi.org/10.1371/journal.pone.0141002

23. Cella M, Knibbe C, de Wildt SN, Van Gerven J, Danhof M, Della Pasqua O (2012) Scaling of pharmacokinetics across paediatric populations: the lack of interpolative power of allometric models. Br J Clin Pharmacol 74:525–535 . https://doi.org/10.1111/j.1365-2125.2012.04206.x

24. Abdelwahab MT, Leisegang R, Dooley KE, Mathad JS, Wiesner L, McIlleron H, Martinson N, Waja Z, Letutu M, Chaisson RE, Denti P (2020) Population Pharmacokinetics of Isoniazid, Pyrazinamide, and Ethambutol in Pregnant South African Women with Tuberculosis and HIV. Antimicrob Agents Chemother 64: . https://doi.org/10.1128/AAC.01978-19

25. Deng R, Gibiansky L, Lu T, Agarwal P, Ding H, Li X, Kshirsagar S, Lu D, Li C, Girish S, Wang J, Boyer M, Humphrey K, Freise KJ, Salem AH, Seymour JF, Kater AP, Miles D (2019) Bayesian Population Model of the Pharmacokinetics of Venetoclax in Combination with Rituximab in Patients with Relapsed/Refractory Chronic Lymphocytic Leukemia: Results from the Phase III MURANO Study. Clin Pharmacokinet 58:1621–1634 . https://doi.org/10.1007/s40262-019-00788-8

26. Magnusson MO, Samtani MN, Plan EL, Jonsson EN, Rossenu S, Vermeulen A, Russu A (2017) Population Pharmacokinetics of a Novel Once-Every 3 Months Intramuscular Formulation of Paliperidone Palmitate in Patients with Schizophrenia. Clin Pharmacokinet 56:421–433 . https://doi.org/10.1007/s40262-016-0459-3

27. Edlund H, Steenholdt C, Ainsworth MA, Goebgen E, Brynskov J, Thomsen OØ, Huisinga W, Kloft C (2017) Magnitude of Increased Infliximab Clearance Imposed by Anti-infliximab Antibodies in Crohn’s Disease Is Determined by Their Concentration. AAPS J 19:223–233 . https://doi.org/10.1208/s12248-016-9989-8

28. Quartino AL, Karlsson MO, Lindman H, Friberg LE (2014) Characterization of endogenous G-CSF and the inverse correlation to chemotherapy-induced neutropenia in patients with breast cancer using population modeling. Pharm Res 31:3390–3403 . https://doi.org/10.1007/s11095-014-1429-9

29. Lledó-García R, Mazer NA, Karlsson MO (2013) A semi-mechanistic model of the relationship between average glucose and HbA1c in healthy and diabetic subjects. J Pharmacokinet Pharmacodyn 40:129–142 . https://doi.org/10.1007/s10928-012-9289-6

30. Stevens J, Ploeger BA, Hammarlund-Udenaes M, Osswald G, van der Graaf PH, Danhof M, de Lange ECM (2012) Mechanism-based PK-PD model for the prolactin biological system response following an acute dopamine inhibition challenge: quantitative extrapolation to humans. J Pharmacokinet Pharmacodyn 39:463–477 . https://doi.org/10.1007/s10928-012-9262-4

31. Kshirsagar SA, Blaschke TF, Sheiner LB, Krygowski M, Acosta EP, Verotta D (2007) Improving data reliability using a non-compliance detection method versus using pharmacokinetic criteria. J Pharmacokinet Pharmacodyn 34:35–55 . https://doi.org/10.1007/s10928-006-9032-2

32. Pérez-Ruixo JJ, Doshi S, Chow A (2011) Application of Pharmacokinetic–Pharmacodynamic Modeling and Simulation for Erythropoietic Stimulating Agents. Clinical Trial Simulations 307–323 . https://doi.org/10.1007/978-1-4419-7415-0_14

33. Cella M, Vries FG de, Burger D, Danhof M, Pasqua OD (2010) A Model-Based Approach to Dose Selection in Early Pediatric Development. Clinical Pharmacology & Therapeutics 87:294–302 . https://doi.org/10.1038/clpt.2009.234
